# Structure-dependent stimulation of gut bacteria by arabinoxylo-oligosaccharides (AXOS): a review

**DOI:** 10.1080/19490976.2024.2430419

**Published:** 2024-11-29

**Authors:** Kai P. Leschonski, Martin S. Mortensen, Lea B.S. Hansen, Kristian B.R.M. Krogh, Mirjam A. Kabel, Martin F. Laursen

**Affiliations:** a National Food Institute, Technical University of Denmark, Kongens Lyngby, Denmark; b Novonesis A/S, Kongens Lyngby, Denmark; c Laboratory of Food Chemistry, Wageningen University, Wageningen, The Netherlands

**Keywords:** Arabinoxylo-oligosaccharides (AXOS), arabinoxylan, microbiome, prebiotics, *Bifidobacterium*, *Bacteroides*, *Prevotella*, Clostridia, Short-chain fatty acids (SCFAs), cross-feeding, metabolism, xylanase

## Abstract

Arabinoxylo-oligosaccharides (AXOS) are non-digestible dietary fibers that potentially confer a health benefit by stimulating beneficial bacteria in the gut. Still, a detailed overview of the diversity of gut bacteria and their specificity to utilize structurally different AXOS has not been provided to date and was aimed for in this study. Moreover, we assessed the genetic information of summarized bacteria, and we extracted genes expected to encode for enzymes that are involved in AXOS hydrolysis (based on the CAZy database). The taxa involved in AXOS fermentation in the gut display a large variety of AXOS-active enzymes in their genome and consequently utilize AXOS to a highly different extent. Clostridia and Bacteroidales are generalists that consume many structurally diverse AXOS, whereas *Bifidobacterium* are specialists that specifically consume AXOS with a low degree of polymerization. Further complexity is evident from the fact that the exact bacterial species, and in some cases even the bacterial strains (e.g. in *Bifidobacterium longum*) that are stimulated, highly depend on the specific AXOS molecular structure. Furthermore, certain species in *Bifidobacterium* and *Lactobacillaceae* are active as cross-feeders and consume monosaccharides and unbranched short xylo-oligosaccharides released from AXOS. Our review highlights the possibility that (enzymatic) fine-tuning of specific AXOS structures leads to improved precision in targeting growth of specific beneficial bacterial species and strains in the gut.

## Introduction

Microorganisms in the human gastrointestinal tract impact both human health and metabolism.^1^ Imbalance of the gut microbiome composition has been associated with the development of various diseases, metabolic health problems, and obesity.^1,2^ Fortunately, the gut microbiome can be positively modulated by promoting the growth of beneficial bacteria through the consumption of prebiotics. Prebiotics are internationally defined as substrates that are selectively utilized by host microorganisms conferring a health benefit and are generally present in the cell walls of fibrous foods, like cereals, fruits, and vegetables.^3^ One of such prebiotic compounds, arabinoxylan (AX), is a major constituent in cereal cell walls, for example, wheat and corn.^4^ A number of studies suggest that arabinoxylan hydrolysis products, i.e., arabinoxylo-oligosaccharides (AXOS), possess an even more potent prebiotic capability compared to non-hydrolyzed AX,^5^ however, an overview of how diverse bacteria can utilize structurally different AXOS is not available, and was aimed at in this study.

The fermentation of AXOS in the gut eventually leads to the formation of beneficial secondary metabolites by a synergistic machinery of bacteria that degrade AXOS directly, and of cross-feeders that utilize the catabolic products released.^6,7^ In this diverse microbial community, different bacteria possess different enzymes and transporters that target specific AXOS structural elements, indicating that the selective stimulation of gut bacteria is highly dependent on the exact AXOS molecular structure.^6–10^ The selective fiber consumption by gut bacteria may be exploited to target the growth of specific, potentially beneficial, strains.^11,12^ The molecular structure of AXOS (i.e., degree, type, and pattern of substitution and degree of polymerization) mainly varies with its source and its extraction method.^4,13^

In this review, we first describe the molecular structure of AX from various cereal sources and tissue types, and the enzymatic degradation of AX resulting in a variety of AXOS structures. The main aim of this review, however, is to provide an overview of how these structurally different AXOS can modulate the gut microbiome. Of summarized bacteria, we assessed their genetic potential and highlighted genes encoding AXOS-degrading enzymes based on the CAZy database. This review gives directions on how (enzymatic) fine-tuning of specific AXOS structures may be a tool to target the growth of specific beneficial bacterial species and strains in the gut, also known as precision prebiotics.

## Arabinoxylan structure and occurrence

Arabinoxylan (AX) is a major polysaccharide present in the cell walls of monocotyledons such as cereals and grasses.^4^ Majorly in their secondary cell wall, cellulose microfibrils are embedded in a matrix of AX esterified to lignin and proteins.^14–17^ AX generally consists of a β-(1→4)-d-xylopyranosyl (Xyl) backbone, with α-l-arabinofuranosyl (Ara) substituents at either *O*-2 and/or *O*-3 positions.^4^ Nevertheless, other substituents can also be present, and the exact composition, and size (molecular weight; Mw), of different AX populations vary between botanical species (e.g., wheat, barley, or corn), tissue type (e.g., endosperm or pericarp), and extraction method (e.g., water or alkali extractable).^4,15,18,19^

**Figure 1.**
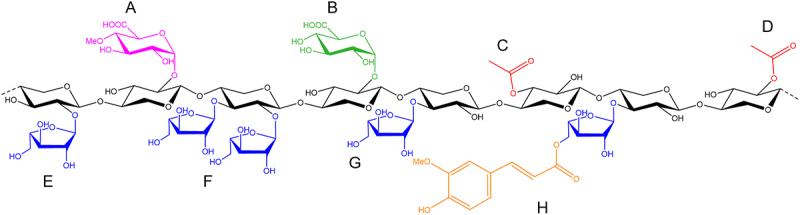
Structural features of arabinoxylan from wheat pericarp cells.^15,20^ a) methylated glucuronic acid, [4-*O*-methyl-α-d-Glc*p*A-(1→2)]-β-d-Xyl*p*. b) glucuronic acid, [α-d-Glc*p*A-(1→2)]-β-d-Xyl*p*. c) acetic acid, 3-*O*-acetyl-β-d-Xyl*p*. d) acetic acid, 2-*O*-acetyl-β-d-Xyl*p*. e) arabinose, [α-l-Ara*f*-(1→2)]-β-d-Xyl*p*. f) arabinose, [α-l-Ara*f*-(1→2)][α-l-Ara*f*-(1→3)]-β-d-Xyl*p*. g) arabinose, [α-l-Ara*f*-(1→3)]-β-d-Xyl*p*. h) ferulic acid, [5-*O*-feruloyl-α-l-Ara*f*-(1→3)]-β-d-Xyl*p*.

In the literature studying prebiotics, wheat AX and its hydrolysis products are most thoroughly examined, and therefore, in this review, wheat AX structures will be described in most detail and compared to AX structures from other cereal sources. In wheat AX, the Xyl backbone is mainly substituted with Ara either as a single substitution at *O*-3 positions (i.e., monosubstituted) or as a double substitution at both *O*-2 and *O*-3 positions (i.e., disubstituted).^4,15^ AX populations from different tissue types predominantly differ in the degree of Ara substitution, and the presence of other less abundant substituents.^15^ For example, AX from the wheat pericarp is more highly substituted with Ara (up to 1.16 Ara/Xyl ratio) compared to the AX from the wheat endosperm (0.84 Ara/Xyl ratio) and aleurone (0.44 Ara/Xyl ratio) cells.^15^ Other substituents that can be present in wheat AX to a lesser extent include Ara monosubstituted at *O*-2 positions (rarely found in wheat), ferulic acid esterified to Ara substituents at *O*-5 (0.3%, 1.2%, and 3.5% (w/w tissue) in endosperm, pericarp, and aleurone, respectively), acetyl esterified to Xyl at *O*-2 and *O*-3 positions (found in pericarp and aleurone), and (4-*O*-methyl-) α-d-glucuronic acid bound to Xyl at *O*-2 positions (found in pericarp) (Figure 1).^15,20^ Ferulic acid substituents can form so-called diferulic bridges to link AX chains together, and ferulic acid can covalently bind with lignin and proteins in the lignocellulose matrix.^14–17^ Therefore, a higher abundance of ferulic acid has been associated with reduced water-solubility of the AX population.^15^ Rye, oat, and barley AX are structurally similar to wheat AX, however, there are notable differences. For example, barley AX has more *O*-2 monosubstituted Xyl,^21^ whereas rye endosperm AX has less disubstituted Xyl,^22^ compared to wheat AX. In oat AX, disubstituted Xyl is absent, and its substituted regions are more clustered compared to the more randomly distributed substituents in wheat AX.^19,23^ Corn AX is more heterogenous and has, in addition to the AX structural elements found in wheat, d-galactopyranosyl (Gal) substituents, and *O*-3 Ara substituents may be further substituted with more Ara, Xyl, and/or d- and l-Gal residues.^24,25^

## Enzymes involved in arabinoxylan degradation

A variety of xylan-hydrolyzing and debranching enzymes, predominantly of fungal and bacterial origin, are known to target the different structural features found in various arabinoxylan (AX) populations (CAZy.org).^26^ Such enzymes may be used as process aid or to target AXOS production, or be part of the enzyme machineries of the bacterial species in our gut. Relevant enzymes include endoxylanases (EC 3.2.1.8 and EC 3.2.1.136), β-xylosidases (BXs) (EC 3.2.1.37), xylobiohydrolases, reducing end xylose-releasing exo-oligoxylanases (Rex) (EC 3.2.1.156), arabinofuranosidases (ABFs) (EC 3.2.1.55), feruloyl esterases (EC 3.1.1.73), α-glucuronidases (EC 3.2.1.139), and acetyl xylan esterases (EC 3.1.1.72) (CAZy.org).^26–28^ In the following sections, we describe the mode-of-actions of endoxylanases, ABFs, BXs, and Rex enzymes active on AX and (A)XOS, based on current literature. Though it should be noted that biochemical analysis and product profiling of AX and (A)XOS active enzymes are still limited, and once an increased number of such enzymes become characterized, their mode-of-action toward various xylan and (A)XOS structures will become more and more targetable.

### Endoxylanases

**Figure 2.**
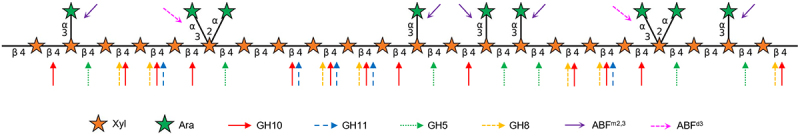
Potential hydrolysis sites in a schematic wheat AX structure by GH5, GH8, GH10, and GH11 endoxylanases and ABF^m2,3^ and ABF^d3^ arabinofuranosidases. The arabinoxylan structure was created with DrawGlycan-SNFG.^33^

Endoxylanases are glycoside hydrolases (GHs) that hydrolyze internal glycosidic linkages of the xylan backbone and are predominantly categorized in so-called GH families 5, 8, 10, 11, and 30 according to the CAZy enzyme database (CAZy.org).^26^ These xylanases have varying specificities and hydrolyze the xylan chain to a different extent depending on the substituents present.^27,29^ Furthermore, the xylanases that hydrolyze AX, accommodate arabinosyl (Ara) substituents at specific subsites in their active site, and therefore will produce AXOS with distinct structures.^27,29–31^ In this review, AXOS structures will be specified following the nomenclature described by Faure et al.^32^ In short, each xylosyl (Xyl) backbone unit (starting from the non-reducing end) is described with either a letter “X” or “A” to indicate the presence of an unsubstituted Xyl unit or a Xyl unit substituted with Ara, respectively. Furthermore, the position of the Ara substituents (i.e., *O*-2 or *O*-3 or both) is indicated with corresponding numbers. For example, 3^2^-α-l-Ara*f*-xylobiose is annotated as A^3^X, and 2^3^,3^3^-di-α-l-Ara*f*-xylotriose is annotated as A^2+3^XX. A schematic of the potential hydrolysis sites of arabinoxylan by endoxylanases and ABFs is displayed in Figure 2.

#### GH10 endoxylanases

When hydrolyzing the AX backbone, GH10 endoxylanases can accommodate Ara substituents at the −3 and +1 subsites of the enzyme and also Ara from *O*-3 monosubstituted Xyl residues at the −2 subsite (Figure 2).^30,34,35^ Thus, from a substantial hydrolysis of wheat AX, these GH10 enzymes predominantly produce the AXOS A^3^X, A^2+3^XX, and A^3^A^3^X.^30,34,35^ There are exceptions and minor differences in the active site structures lead to a slightly different affinity for the various substituted Xyl residues among different GH10 enzymes, thus resulting in slightly different product profiles.^30^ For instance, some GH10 endoxylanases have been shown to also produce AXOS resulting from binding of Ara substituents in the +2 subsite of the enzyme (i.e., XA^3^X, XA^3^A^3^X, XA^2+3^XX) besides the above-mentioned products.^30,35–37^ From unsubstituted regions in the Xyl backbone, these GH10 enzymes release linear xylo-oligosaccharides (XOS), which, if consisting of at least three Xyl units, can be further hydrolyzed to XOS consisting of at least two Xyl units and even xylose.^29,30,38^

#### GH11 endoxylanases

GH11 endoxylanases accommodate Ara substituents in the −3 and +2 subsites when acting on AX, and thus produce predominantly the AXOS XA^3^XX, XA^2+3^XX, and XA^3^A^3^XX from wheat AX (Figure 2).^30,34,39–41^ Furthermore, these GH11 enzymes release linear XOS from unsubstituted regions in the xylan backbone, which, if consisting of at least four Xyl units, are further hydrolyzed to XOS consisting of at least two or three Xyl units and/or xylose.^30,42^

#### GH8 endoxylanases

GH8 endoxylanases have not yet been thoroughly researched, and currently only 10 have been characterized in CAZy (CAZy.org).^26^ Although only a few product profiles of GH8 endoxylanases on AX have been described, currently characterized GH8s can tolerate Ara substituents in the +2 subsite of the enzyme, and from wheat AX, the major monosubstituted AXOS produced includes XA^3^X, whereas the major disubstituted AXOS produced includes XA^2+3^XXX (Figure 2).^42–44^ Possibly, GH8 enzymes are more hindered by disubstituted Xyl compared to monosubstituted Xyl, though more enzymes need to be characterized to confirm their hydrolysis sites toward various AX structures. From unsubstituted regions in AX, GH8 endoxylanases release linear XOS, of which predominantly XOS consisting of at least six Xyl units are further hydrolyzed to XOS consisting of at least two to five Xyl units.^42–44^ Low affinity toward XOS consisting of five or less Xyl units by currently characterized GH8 endoxylanases could be attributed to a long substrate-binding cleft that is comprised of six subsites.^43–45^

#### GH5 endoxylanases

GH5 endoxylanases have also not yet been thoroughly examined, and the AXOS these enzymes produce from AX have only been identified for four GH5 enzymes (in subfamily 34).^31^ Currently characterized GH5 xylanases are not active toward linear xylan, and instead cleave the xylan chain after binding of an Ara substituent (in the −2* pocket of the active site) monosubstituted to a Xyl located in the −1 subsite of the enzyme.^31,46–49^ The major hydrolysis products GH5 endoxylanases produce from wheat AX include AXOS substituted with Ara on the non-reducing end Xyl (Figure 2).^31,46–49^ Whether Ara from disubstituted Xyl residues in AX allow for binding and subsequent hydrolysis of the xylan chain by GH5 xylanases has not yet been investigated.

#### GH30 endoxylanases

GH30 xylanases, another relatively newly discovered group of enzymes, can be found in subfamilies GH30_7 (fungal) and GH30_8 (bacterial).^50,51^ These enzymes are often described as glucuronoxylanases, though, depending on their active site structure GH30 xylanases may show diverse substrate specificities.^50,51^ The active site structure of currently characterized bacterial GH30_8 xylanases is highly conserved among most enzymes, and they require α-1,2-linked methylated glucuronic acid (MeGlcA) residues for binding and subsequent hydrolysis of the xylan backbone.^50,52,53^ Though one GH30_8 from *Clostridium acetobutylicum* has been reported to also hydrolyze endo-β-1,4 xylosidic linkages from linear xylan and XOS (DP > 4) and from substituted arabinoxylan, besides the expected MeGlcA-specific endoxylanase activity.^53^ The GH30_7 subfamily shows more diverse substrate specificity and, besides endoxylanase, also comprises xylobiohydrolase and Rex activities.^51^ Furthermore, the fungal GH30_7 xylanases are less strictly dependent on MeGlcA residues compared to GH30_8 xylanases, and, varying among enzymes, these GH30_7 xylanases may not require substituents to hydrolyze the xylan backbone, and they may also bind non-methylated GlcA residues for the subsequent hydrolysis of the xylan backbone.^50^ Arabinoxylans are still poor substrates for most characterized GH30_7 endoxylanases and are barely hydrolyzed,^50,51^ though one GH30_7 from *Talaromyces cellulolyticus* (TcXyn30C) with a slightly different active site structure compared to other GH30_7s has been shown to bind Ara substituents in the −2 subsite for xylanase activity on AX.^54^

### Arabinofuranosidases

Arabinofuranosidases (ABFs) are GHs that include the enzymes that hydrolyze Ara substituents from AX and AXOS, and are mainly categorized in GH families 43, 51, 54, and 62 (CAZy.org).^26^ Most ABFs hydrolyze Ara exclusively from monosubstituted Xyl (ABF^m2,3^),^27^ nevertheless, four candidates within GH43 (two fungal in GH43_36 and two bacterial in GH43_10) have been found to hydrolyze α-(1→3)-Ara from disubstituted Xyl (ABF^d3^) (Figure 2).^27,28,55–58^ Moreover, some ABF^m2,3^ candidates within GH51 have been found to hydrolyze Ara from disubstituted AXOS exclusively when disubstituted on the non-reducing end Xyl.^55,56^

### β-xylosidases and exo-xylanases

After ABF and xylanase action, β-xylosidases (BXs) hydrolyze Xyl units from the non-reducing end of xylo-oligosaccharides (EC 3.2.1.37). BXs are GHs predominantly found in GH families 3, 39, 43, 52, and 120 (CAZy.org).^26,27^ Interestingly, a relatively newly discovered group of enzymes has been found to hydrolyze Xyl units from the reducing end of XOS (EC 3.2.1.156), so-called reducing end xylose-releasing exo-oligoxylanases (Rex), seven bacterial in GH8 and two fungal in GH30 (CAZy.org).^26,27,59,60^ Nevertheless, more Rex enzymes need to be characterized to determine their specificities and binding affinities toward various substrate structures.

### Impact of AXOS on the gut microbiome composition

Substituted xylo-oligosaccharides (XOS) are non-digestible by humans and thus reach the colon intact.^61^ To ferment such oligosaccharides, due to their recalcitrant nature (β-(1→4)-xylopyranosyl backbone and heterogenous substituent composition), gut bacteria require specific enzymes (either intra- and/or extracellular) and targeted transporters.^10,62,63^ Thereby, depending on the oligosaccharide structures and bacteria present in the gut, specific bacteria are stimulated.^62,63^ Generally, the prebiotic effect of substituted XOS is mainly dependent on their size, i.e., degree of polymerization (DP), followed by the substitution pattern.^61,64–66^ Furthermore, lower DP and less substituted XOS are generally fermented faster, whereas higher DP and more substituted XOS reach further in the colon.^64,66^ Here, we first reviewed the impact of arabinoxylo-oligosaccharides (AXOS) on the growth of diverse gut bacteria, including the formed short-chain fatty acids and other metabolites. The results were compiled in Tables 1 and 2.

**Table 1.** Overview of significant changes in bacterial compositions observed in complex gut microbial communities (microbiomes) after supplementation of partially hydrolyzed arabinoxylan and AXOS, and the associated metabolites released. The AXOS source, hydrolysis method used, size (DP) and presence of substituents was specified if possible. *In vitro* human fecal culture studies analyzed human fecal cultures that grew on AXOS. *In vivo* human studies contain directly analyzed fecal compositions from humans that ingested AXOS. Metabolite concentrations from highest timepoints were annotated if possible: + (<2 µm), ++ (>3 µm), +++ (>15 µm), ++++ (>25 µm).

Study	Supplement	Change in bacterial composition	Metabolites	Source
*In vitro* human fecal culture	Wheat bran (hydrothermal, DP 5-19)	**Increased**: *Bifidobacterium* spp.^1^	Acetate ++++ Butyrate ++ Propionate ++ Lactate^2^ +	67
*In vitro* human fecal culture	Corn straw (hydrothermal, avDP 4-6)	**Increased**: *Bifidobacterium* spp. *Prevotella* spp. and/or *Bacteroides* spp.	Acetate ++++ Propionate ++ Butyrate ++ Lactate^2^ +	68
*In vitro* human fecal culture	Corn straw (hydrothermal, avDP 9-20)	**Increased**: *Bifidobacterium* spp. *Prevotella* spp. and/or *Bacteroides* spp.	Acetate ++++ Propionate +++ Butyrate ++ Lactate^2^ +	68
*In vitro* human fecal culture	Brewer’s spent grain (GH10, DP 2-16)	**Increased**: *Bifidobacterium* spp. *Lactobacillaceae*^3^ *Prevotella* spp. and/or *Bacteroides* spp.	Acetate ++++ Butyrate +++ Propionate ++ Formate + Lactate^2^ +	69
*In vitro* human fecal culture	Wheat distillers’ dried grains (GH11, DP ≥ 3)	**Increased**: *Bifidobacterium* spp.	Acetate ++++ Propionate ++ Lactate^2^ + Formate^2^ +	62
*In vitro* human fecal culture	A^2^XX	**Increased**: *Bifidobacterium* spp.^1^	Acetate +++ Propionate ++ Butyrate ++	8
*In vitro* human fecal culture	A^2+3^XX	**Increased**: *Bifidobacterium* spp.^1^	Acetate ++++	8
*In vitro* human fecal culture	Arabinoxylan (hydrolyzed, avDP 6, DS 0.38)	**Increased**: Donor 1 *Bacteroides* spp. *Prevotella* spp. *Bifidobacterium* spp. *Roseburia* spp. *Anaerostipes* spp. *Erysipelotrichaceae* (uncl.) Donor 2 *Bacteroides* spp. *Oscillospira* spp. *Megamonas* spp. Donor 3 *Prevotella* spp. *Bifidobacterium* spp. *Enterococcus* spp. *Erysipelotrichaceae* (uncl.) *Blautia* spp. *Veillonella* spp. **Reduced**: In all donors *Anaerotruncus* spp. *Flavonifractor* spp. *Lachnospira* spp. *Parasutterella* spp. Donor 1 *Faecalibacterium* spp. *Escherichia-Shigella* spp. *Lachnospiraceae* (uncl.) Donor 2 *Escherichia-Shigella* spp. *Lachnospiraceae* (uncl.) *Ruminococcaceae* (uncl.) *Subdoligranulum* spp. Donor 3 *Ruminococcaceae* (uncl.) *Subdoligranulum* spp.	Acetate +++ Propionate ++ Butyrate ++	70
*In vitro* human fecal culture	Brewer’s spent grain (hydrothermal, DP 2-10)	n.d.	Donor 1: Acetate ++ Propionate ++ Lactate^2^ + Donor 2: Acetate ++ Propionate + Butyrate + Lactate^2^ +	61
*In vitro* human fecal culture	Acetylated XOS from brewer’s spent grain (hydrothermal, DP 2-10)	n.d.	Donor 1: Acetate +++ Lactate +++ Propionate ++ Donor 2: Acetate ++ Butyrate ++ Lactate^2^ +	61
*In vitro* human fecal culture	Feruloylated AXOS from wheat bran (xylanase, avDP 4.55)	**Increased**: *Bifidobacterium* spp. *Faecalibacterium* spp. *Acidaminococcus* spp. *Dialister* spp. *Megasphaera* spp. *Megamonas* spp. **Reduced**: *Alistipes* spp. *Bacteroides* spp. *Bilophila* spp. *Blautia* spp. *Coprococcus* spp. *Desulfovibrio* spp. *Dorea* spp. *Enterobacteriaceae* spp. *Oscillospira* spp. *Parabacteroides* spp. *Phascolarctobacterium* spp. *Ruminococcus* spp.	Acetate ++ Propionate ++ Butyrate +	71
*In vivo* human	Wheat bran extract enriched in AXOS	**Increased**: *Bifidobacterium adolescentis* *Bifidobacterium longum* *Bifidobacterium catenulatum* *Bifidobacterium angulatum* *Prevotella* spp. *Prevotella copri* *Ruminococcus gnavus* *Lachnospiraceae* (uncl.) **Reduced**: *Rikenella* spp. *Parabacteroides* spp. *Paraprevotella* spp.	Acetate Propionate Succinate	72
*In vivo* human	Wheat bran (hydrolyzed, avDP 3-8)	**Increased**: *Bifidobacterium* spp. *Akkermansia* spp. *Prevotellaceae* (uncl.) *Lactobacillaceae*^3^ **Reduced**: *Blautia* spp. *Anaerobutyricum hallii* *Coriabacteriaceau* (uncl.) *Dorea* spp.	No change^4^	73
*In vivo* human	Wheat bran (hydrolyzed, avDP 5)	**Increased**: *Bifidobacterium longum* *Bifidobacterium adolescentis* *Prevotella copri*^5^ *Bacteroides uniformis*^5^ *Bacteroides cellulosilyticus*^5^ **Reduced**: *Bacteroides* spp.^5^	No change^4^	74
*In vivo* human	Wheat bran (GH11, avDP 6, DS 0.26)	**Increased**: *Bifidobacterium* spp. (including *B. adolescentis*) **Reduced**: *Lactobacillaceae*^3^	No change^4^	75
*In vivo* human	Wheat bran (hydrolyzed, avDP 5, DS 0.24)	**Increased**: *Bifidobacterium longum* *Bifidobacterium adolescentis* *Ruminococcus obeum* *Agathobacter rectalis* *Faecalibacterium prausnitzii* *Dorea longicatena* *Blautia wexlerae* *Fusicantenibacter saccharivorans* **Reduced**: *Clostridium methylpentosum* *Anaerotruncus colihominis* *Erysipelothrix rhusiopathiae*	n.d.	76
*In vivo* human	Bread with added xylanase (avDP 18)	No change	Butyrate ++	77
*In vivo* human	Wheat bran (GH11, avDP 5, DS 0.19)	**Increased**: *Bifidobacterium* spp.	Acetate ++++ Propionate ++ Butyrate ++	78,79
*In vivo* human	Bread with added xylanase (avDP 18)	**Increased**: *Lactobacillaceae*^3^ & *Enterococcus* spp. *Bacteroides* spp. & *Prevotella* spp.	Acetate Butyrate Propionate	80
*In vivo* human	Wheat bran (GH11, avDP 5, DS 0.22)	**Increased**: *Bifidobacterium adolescentis*^1^	n.d.	81
*In vivo* human	Wheat bran in cereal (hydrolyzed)	**Increased**: *Bifidobacterium* spp.	n.d.	82
*In vivo* human	Wheat bran in bread (hydrolyzed)	n.d.	Acetate ++++ Butyrate +	83
*In vitro* simulated colon	Wheat bran (GH11, avDP 29, DS 0.30)	**Increased**: *Lactobacillaceae*^3^ *Bifidobacterium* spp. Clostridia *Bacteroides* spp. and/or *Prevotella* spp. *Selenomonas* spp.	Butyrate ++ Propionate ++ Acetate ++	84
*In vitro* simulated colon	Wheat bran (GH11)	**Increased**: *Agathobacter* spp. *Megaspaera* spp. *Lachnoclostridium* spp. *Bacteroides* spp. **Reduced**: *Veillonella parvula* *Enterococcus faecalis* *Aeromonas caviae* *Bucella* spp. *Ochrobactrum* spp.	Acetate ++++ Propionate ++ Butyrate ++	85
*In vitro* simulated colon	Wheat bran (GH10 & GH11, avDP 74, DS 0.61) (GH10 & GH11, avDP 46, DS 0.63) (GH10 & GH11, avDP 42, DS 0.92) (GH10 & GH11, avDP 40, DS 0.34) (GH10 & GH11, avDP 4, DS 0.27)	n.d.	Acetate +++ Propionate +++ Butyrate +	66
*In vitro* simulated colon	Wheat bran (GH11, avDP 6.3, DS 0.36)	**Increased**: *Bifidobacterium* spp.	Acetate +++ Propionate ++ Butyrate +	86
*In vitro* simulated colon	Feruloylated AXOS from wheat bran (GH11, avDP 6.6, DS 0.38)	**Increased**: *Bifidobacterium* spp.	Acetate +++ Propionate ++ Butyrate +	86
*In vivo* rats	Wheat bran (GH10 & GH11, avDP 3, DS 0.26) (GH10 & GH11, avDP 5, DS 0.27)	**Increased**: *Bifidobacterium* spp.	Acetate Butyrate	64
*In vivo* rats	Wheat bran (GH10, avDP 12, DS 0.69)	No change	No change	64
*In vivo* rats	Wheat bran (GH11, avDP 15, DS 0.27)	No change	No change	64
*In vivo* rats	Wheat bran (GH11, avDP 5, DS 0.24)	**Increased**: *Bifidobacterium* spp.	Acetate Propionate Butyrate	87
*In vivo* mice	Wheat bran (hydrolyzed)	**Increased**: *Bifidobacterium* spp.	n.d.	88
*In vivo* chickens	Wheat bran (xylanase, avDP 9, DS 0.34) (xylanase, avDP 3, DS 0.09)	**Increased**: *Bifidobacterium* spp.	n.d.	89
*In vivo* chickens	Wheat bran (GH11, avDP 15, DS 0.27)	**Increased**: *Bifidobacterium* spp.^1^	n.d.	90
*In vitro* chicken cecal culture	Wheat bran (GH11, avDP < 10)	**Increased**: *Faecalibacterium* spp. *Intestinimonas* spp. **Reduced**: *Bacteroides* spp.	Butyrate ++ Acetate ++ Propionate ++	91
*In vivo* sturgeons	Wheat bran (GH11, avDP 3, DS 0.25)	**Increased**: *Clostridium ruminantium* *Lactococcus* sp. *Eubacterium* spp.	No change	65,92
*In vivo* sturgeons	Wheat bran (GH11, avDP 32, DS 0.30)	**Increased**: *Bacillus circulans* *Bacillus clausii* *Clostridium* spp. *Clostridium baratii* *Clostridium colicanis* *Lactococcus lactis* *Lactobacillaceae*^3^ *Ligilactobacillus aviarius* *Eubacterium* spp. *Eubacterium budayi*	Acetate Butyrate	65,92

^1^Study did not investigate other bacteria that may be present. ^2^Only present during initial fermentation times.^3^*Lactobacillus* spp. was recently reclassified into many different genera in the *Lactobacillaceae* family.^4,93^ Likely not detected due to uptake of metabolites by epithelial cells in the gut. ^5^Donor dependent. (av)DP: (Average) degree of polymerization. DS: Degree of substitution. n.d.: Not determined.

**Table 2.** Overview of bacterial strains with observed growth on AXOS in *in vitro* single/co-culture growth studies. The substrate source, enzymes used, size (DP), and presence of substituents in the substrate and substrate concentration (mg/mL) was specified if possible. Growth was quantified if possible as **∆**OD_600_: 0–0.10 (no growth), 0.11–0.39 (poor growth), 0.40–0.59 (medium growth), 0.60–0.79 (good growth), >0.8 (high growth). Metabolites concentrations from the highest time points were annotated if possible: + (<3 mM), ++ (4–9 mM), +++ (10–15 mM), ++++ (>16 mM).

Bacteria	Substrate	Conc. (mg/mL)	Time (h)	∆OD_600_	Metabolites released	Source
*Bifidobacterium adolescentis* ATCC 15703	AXOS from wheat (avDP 5, DS 0.27)	5	4	0.1	n.d.	63
			8	0.55		
			12	0.6		
			24	0.6		
			48	0.6		
	AXOS from wheat (GH10)	4	60	0.1-0.39	Acetate	8
			140	0.40-0.69		
	AXOS from wheat (GH10)	5	5	0.1	n.d.	94
			10	0.85		
			15	0.80		
	AXOS from wheat (GH5 + ABF^d3^)	5	12	0.2-0.3	Acetate ++ Lactate + Propionate* +	95
			24	0.3-0.4		
	AXOS from wheat (GH11, DP 2-3)	5	24	1	Acetate ++++ Lactate + Propionate* ++ Butyrate* +	96
	AXOS from wheat (GH11, DP 2-6)	5	24	0.8	Acetate ++ Lactate + Propionate* ++ Butyrate* +	
	AXOS from wheat (GH11, feruloylated)	5	24	0.8	Acetate ++ Lactate ++ Propionate* ++ Butyrate* +	
	AXOS from wheat (GH11)	5	24	0.2-0.3	Acetate ++++ Lactate +++	97
			48	0.2-0.3		
	AXOS from wheat (GH11, purified)	5	48	n.d.	n.d.	98
	AXOS from rye (GH10)	4	60	0.1-0.39	Acetate	8
			140	0.1-0.39		
	AXOS from rye (GH10)	5	24	n.d.	n.d.	99
			48			
			72			
	AXOS from rye (GH5)	5	24	0.89	n.d.	47
			48	0.92		
	Water soluble AXOS from barley (GH11)	5	48	0.19	Acetate ++++ Lactate +++ Propionate* +++ Butyrate* +	100
	Alkali soluble AXOS from barley (GH11)	5	48	0.13	Acetate ++++ Lactate +++ Propionate* +++ Butyrate* +	
	Water soluble AXOS from barley (GH11, BSG)	5	48	0.14	Acetate ++++ Lactate +++ Propionate* +++ Butyrate* +	
	Alkali soluble AXOS from barley (GH11, BSG)	5	48	0.13	Acetate ++++ Lactate +++ Propionate* +++ Butyrate* +	
*Bifidobacterium adolescentis* B72	AXOS from wheat (avDP 5, DS 0.27)	5	4	0.6	Acetate ++++ Lactate ++++	101
			8	1.05		
			12	1.05		
			24	1.05		
			48	1.1		
*Bifidobacterium catenulatum* LMG 11043	AXOS from wheat (avDP 5, DS 0.27)	5	4	0	n.d.	63
			8	0.3		
			12	0.75		
			24	0.75		
			48	1		
*Bifidobacterium pseudocatenulatum* YIT 4072	AXOS (GH10)	5	5	0	n.d.	10
			10	0.05		
			15	0.3		
			20	0.6		
			25	0.6		
*Bifidobacterium longum* ATCC 15707	AXOS from wheat (avDP 5, DS 0.27)	5	4	0.25	n.d.	63
			8	0.5		
			12	0.5		
			24	0.55		
			48	0.6		
	AXOS from wheat (GH10)	4	60	0.1-0.39	Acetate	8
			140	0.1-0.39		
	AXOS from rye (GH10)	4	60	0.1-0.39	Acetate	8
			140	0.1-0.39		
	AXOS from wheat (GH11, purified)	5	48	n.d.	n.d.	98
	A^2+3^XX	10	12	0.05	n.d.	102
			24	0.2		
			36	0.5		
			48	0.5		
*Bifidobacterium longum* CUETM 172	AXOS from wheat (avDP 5; DS 0.27)	5	4	0.05	n.d.	63
			8	0.1		
			12	0.15		
			24	0.3		
			48	0.25		
*Bifidobacterium longum* subsp. *longum* 46	AXOS from wheat (avDP 5, DS 0.27)	5	4	0.15	n.d.	63
			8	0.45		
			12	0.55		
			24	0.55		
			48	0.55		
*Bifidobacterium longum* subsp. *longum* B24	AXOS from wheat (avDP 5, DS 0.27)	5	4	0.15	Acetate ++ Lactate ++ Formate ++	101
			8	0.25		
			12	0.35		
			24	0.4		
			48	0.4		
*Bifidobacterium longum* subsp. *longum* B18	AXOS from wheat (avDP 5, DS 0.27)	5	4	0.3	Acetate ++++ Lactate +++ Formate ++	101
			8	0.55		
			12	0.65		
			24	0.75		
			48	0.85		
*Bifidobacterium longum* subsp. *longum* PRO 16-10	AXOS from wheat (avDP 5, DS 0.27)	5	4	0.05	n.d.	63
			8	0.15		
			12	0.55		
			24	0.55		
			48	0.55		
*Bifidobacterium longum* subsp. *longum* CUETM 290	AXOS from wheat (avDP 5, DS 0.27)	5	4	0.05	n.d.	63
			8	0.1		
			12	0.15		
			24	0.15		
			48	0.15		
*Bifidobacterium longum* subsp. *longum* CUETM 171	AXOS from wheat (avDP 5, DS 0.27)	5	4	0.3	n.d.	63
			8	0.5		
			12	0.5		
			24	0.6		
			48	0.75		
*Bifidobacterium longum* subsp. *longum* BB536	AXOS from wheat (avDP 5, DS 0.27)	5	4	0.1	n.d.	63
			8	0.25		
			12	0.3		
			24	0.4		
			48	0.45		
*Bifidobacterium longum* subsp. *longum* CUETM 193	AXOS from wheat (avDP 5, DS 0.27)	5	4	0.2	n.d.	63
			8	0.55		
			12	0.7		
			24	0.9		
			48	0.9		
*Bifidobacterium longum* subsp. *longum* LMG 11047	AXOS from wheat (avDP 5, DS 0.27)	5	4	0.2	n.d.	63
			8	0.55		
			12	0.6		
			24	0.8		
			48	0.8		
*Bifidobacterium longum* subsp. *longum* CUETM 239	AXOS from wheat (avDP 5, DS 0.27)	5	4	0.15	n.d.	63
			8	0.55		
			12	0.7		
			24	0.75		
			48	0.9		
*Bifidobacterium longum* subsp. *longum* NCC2705	AXOS from wheat (avDP 5, DS 0.27)	5	4	0.1	n.d.	63
			8	0.35		
			12	0.5		
			24	0.45		
			48	0.55		
	AXOS from wheat (avDP 5, DS 0.27)	5	3	0.3	Acetate ++++ Formate ++ Lactate + Ethanol +	103
			6	0.6		
			9	0.7		
			12	0.7		
			24	0.8		
			48	0.8		
	A^2+3^XX	10	12	0.0	n.d.	102
			24	0.05		
			36	0.1		
			48	0.15		
*Bifidobacterium longum* subsp. *longum* NCC2705 *&* *Agathobacter rectalis* ATCC 33656	AXOS from wheat (avDP 5, DS 0.27)	5	3	1.05	Acetate ++++ Butyrate +++ Formate +++ Lactate ++ Ethanol ++	103
			6	2.25		
			9	2.2		
			12	2.15		
			24	1.85		
			48	1.25		
*Agathobacter rectalis* ATCC 33656	AXOS from wheat (avDP 5, DS 0.27) & added acetate	5	3	0.5	Butyrate ++++ Lactate ++	103
			6	1.5		
			9	1.55		
			12	1.45		
			24	1.35		
			48	1.25		
*Roseburia hominis* DSM 16839	AXOS from wheat (GH5 + ABF^d3^)	5	12	0.4-0.5	Butyrate +++ Lactate ++ Formate +	95
			24	0.6-0.8		
*Faecalibacterium duncaniae* DSM 17677**	AXOS from wheat (GH11, DP 2-3)	5	24	1	Lactate ++ Propionate* +++	96
	AXOS from wheat (GH11, DP 2-6)	5	24	0.8	Lactate + Acetate* ++ Propionate* ++	
	AXOS from wheat (GH11, feruloylated)	5	24	0.8	Butyrate +++ Lactate + Acetate* ++++ Propionate* ++++	
*Prevotella copri* DSM 18205	AXOS from wheat (GH11, DP 2-3)	5	24	0.98	Acetate ++++ Propionate ++ Lactate ++	96
	AXOS from wheat (GH11, DP 2-6)	5	24	0.82	Acetate +++ Propionate ++ Lactate + Butyrate* +	
	AXOS from wheat (GH11, feruloylated)	5	24	0.79	Propionate +++ Acetate ++ Lactate +	
	AXOS from barley (BSG, GH11)	25	48	n.d.	n.d.	104
			72			
*Prevotella ruminicola* 23	AXOS from corn (GH10, GH3, GH51, & GH43_36, acetylated and feruloylated, purified)	30	0	0	n.d.	105
			5	0		
			10	0.27		
			15	0.64		
*Phocaeicola vulgatus* ATCC 8482	AXOS from wheat (GH11)	5	48	n.d.	n.d.	98
*Bacteroides cellulosilyticus* DSM 14838	A^3^X	5	n.r.	0.57	n.d.	106
	A^2^XX			0.71		
	XA^3^XX			0.66		
	A^2+3^XX			0.71		
	A^2^XX/A^3^XX			0.76		
	XA^2+3^XX			0.52		
	XA^2^XX/XA^3^XX			0.62		
*Bacteroides ovatus* ATCC 8483	AXOS from wheat (GH11)	5	48	n.d.	n.d.	98
	A^3^X	5	n.r.	0.75	n.d.	106
	A^2^XX			0.55		
	XA^3^XX			0.7		
	A^2+3^XX			0		
	A^2^XX/A^3^XX			0.66		
	XA^2+3^XX			0.52		
	XA^2^XX/XA^3^XX			0.58		
*Bacteroides ovatus* 3-1-23	A^3^X	5	n.r.	0.83	n.d.	106
	A^2^XX			0.75		
	XA^3^XX			0.8		
	A^2+3^XX			0.5		
	A^2^XX/A^3^XX			0.74		
	XA^2+3^XX			0.6		
	XA^2^XX/XA^3^XX			0.62		
*Bacteroides eggerthii* DSM 20697	A^3^X	5	n.r.	0.32	n.d.	106
	A^2^XX			0.44		
	XA^3^XX			0.42		
	A^2+3^XX			0.47		
	A^2^XX/A^3^XX			0.33		
	XA^2+3^XX			0.35		
	XA^2^XX/XA^3^XX			0.39		
*Bacteroides intestinalis* DSM 17393	A^3^X	5	n.r.	0.57	n.d.	106
	A^2^XX			0.63		
	XA^3^XX			0.43		
	A^2+3^XX			0.53		
	A^2^XX/A^3^XX			0.65		
	XA^2+3^XX			0.34		
	XA^2^XX/XA^3^XX			0.62		
*Bacteroides xylanisolvens* DSM 18836	A^3^X	5	n.r.	0.99	n.d.	106
	A^2^XX			0.93		
	XA^3^XX			0.74		
	A^2+3^XX			0.79		
	A^2^XX/A^3^XX			0.98		
	XA^2+3^XX			0.67		
	XA^2^XX/XA^3^XX			0.77		
*Bacteroides thetaiotaomicron* ATCC 29148	A^3^X	5	n.r.	0.58	n.d.	106
	A^2^XX			0		
	XA^3^XX			0		
	A^2+3^XX			0		
	A^2^XX/A^3^XX			0		
	XA^2+3^XX			0		
	XA^2^XX/XA^3^XX			0		

*The strain does not possess the genes to produce the specific SCFA.^107–109^ **Strain does not possess AXOS-degrading enzymes in its genome (CAZy.org).^26^ BSG: Brewer’s spent grains. n.d.: Not determined. n.r.: Not reported. (av)DP: (Average) degree of polymerization. DS: Degree of substitution.

**Figure 3.**
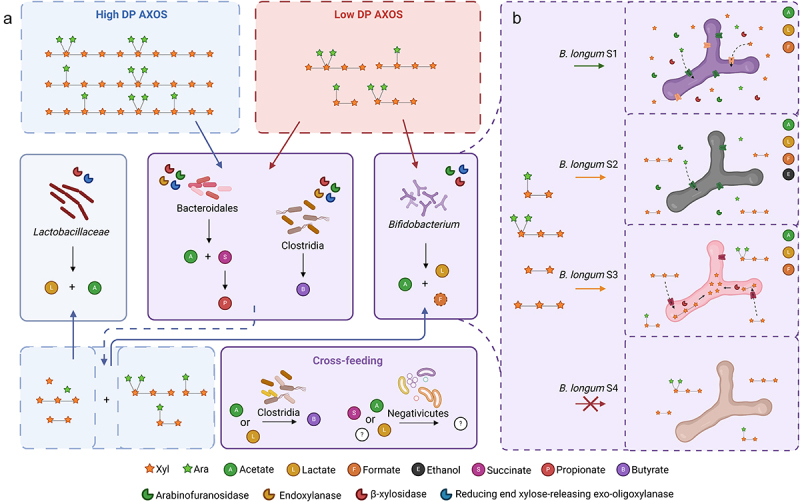
Illustration showing the fermentation of AXOS and associated (simplified) cross-feeding mechanisms in the gut. a) high DP AXOS (blue) are degraded by Bacteroidales and clostridia, and the released hydrolysis products can be utilized by either *bifidobacterium* or *Lactobacillaceae*. Low DP AXOS (red) are utilized by *Bifidobacterium*, Bacteroidales, and clostridia. Cross-feeding clostridia utilize the metabolites acetate and lactate to produce butyrate, and negativicutes utilize metabolites with varying metabolisms. b) the AXOS utilization by *B. longum* is strain-dependent (may not be limited to this genus and species), as different strains possess different enzymes and specific transporters. Furthermore, some *B. longum* strains produce additional acetate, formate, and potentially ethanol instead of lactate when fermenting AXOS. Created with BioRender.com.

Generally, five major groups of microorganisms were distinguished within the human gut microbiota that benefit from AXOS: *Bifidobacterium*, *Lactobacillaceae*, Bacteroidales, Clostridia, and Negativicutes (Tables 1 and 2, Figure 3). The stimulation of the pronounced bacterial groups appears to vary among studies and individuals, but also depends on the AXOS supplemented. Bacteroidales and Clostridia are stimulated by AXOS regardless of DP, whereas *Bifidobacterium* is stimulated by low DP and *Lactobacillaceae* is stimulated by high DP AXOS (Tables 1 and 2, Figure 3). These bacteria release various metabolites from the fermentation of AXOS, which may be utilized by cross-feeding Clostridia and Negativicutes (Tables 1 and 2, Figure 3). Still, it remains elusive which exact bacterial species and strains are competitive in the gut microbiome community, which is expected to be dependent on their specific enzymes and sugar transporters. Unfortunately, knowledge on (presence and type of) transporters is so far very limited.^110–112^ Nevertheless, helped by the huge amount of information currently available from many bacterial genomes (Figure S1-S6) (CAZy.org),^26^ in the next sections we provide detailed information for the found AXOS-active taxa and their corresponding genes predicting AXOS-degrading CAZymes. A schematic summary is shown in Figure 3.

### Bifidobacterium

*Bifidobacterium* is the bacterial genus most often reported to be stimulated by AXOS in complex gut microbiome communities, predominantly by lower DP AXOS (DP < ~10) (Table 1).^64,89^ On a species level mainly *B. adolescentis* and *B. longum* were significantly increased by AXOS (Table 1),^72,74–76,81^ whereas *B. catenulatum* and *B. angulatum* were also significantly increased, though only in one study.^72^

#### GH gene profiles within Bifidobacterium

The growth of *Bifidobacterium* on (low DP) AXOS is expected because many *Bifidobacterium* strains, including *B. adolescentis* and *B. longum* strains, possess many β-xylosidase (BX) encoding genes predominantly in GH3, GH43, and GH120 and arabinofuranosidase (ABF) encoding genes in GH43 and GH51 (Figure S1).^26^ Some strains, for example, *B. adolescentis* ATCC 15703 and *B. pseudocatenulatum* YIT 4072, even possess ABFs that target disubstituted AXOS (in GH43_10) and reducing end xylose-releasing exo-oligoxylanases (Rex) (in GH8) (Figure S1).^10,26,63^ Still, being present in the genome does not guarantee bacterial production of corresponding enzymes, and therefore, their presence, and sometimes their number, is mainly considered indicative.

*Bifidobacterium* strains generally do not possess endoxylanase encoding genes, though some strains in *B. pseudocatenulatum*, *B. catenulatum* subsp. *kashiwanohense*, *B. animalis* subsp. *lactis*, and *B. imperatoris* possess a GH10 encoding gene (Figure S1).^26^ Therefore, utilization of higher DP AXOS and AX likely requires “help” from bacteria that produce extracellular xylanases, as found in, for example, Clostridia and Bacteroidales.^5,94,113^ Indeed, *Bifidobacterium* has been found to be co-stimulated together with Bacteroidales and/or Clostridia from higher DP AXOS in multiple studies (Table 1).^68,69,84^

Within *Bifidobacterium*, not only the types of genes define their GH gene profiles but their total number of GH genes as well. Taken the latter into account, these bifidobacteria “cluster” in several groups that are expected to utilize AXOS to a highly different extent (Figure S1).^26^ One group, with *B. adolescentis*, *B. catenulatum*, and *B. pseudocatenulatum*, possesses a large amount and variety of genes encoding AXOS-degrading enzymes and is expected to grow well on AXOS. The second group, with *B. angulatum*, *B. animalis*, *B. dentium*, and *B. pseudolongum*, has relatively few genes encoding AXOS-degrading enzymes, whereas a third group, with *B. bifidum* and *B. breve*, barely has any. Besides the proposed division into these three groups, which is merely a guideline rather than a strict division due to interspecies variety, another separate group, which comprises the *B. longum* species comes forth. Exclusively in the *B. longum* species even different strains show a large variety in their genetic GH profiles relevant for the degradation of AXOS (Figure S1), in which some *B. longum* strains possess few, and others many GH encoding genes. Fittingly, the AX-hydrolysis product utilization mechanisms in *B. longum* appear to be strain-dependent as well.^63,101,103^

#### Strain-dependent AXOS utilization by B. longum

In line with the strain-dependent GH gene profile found in *B. longum*, various *B. longum* strains utilize different AX hydrolysis products to a highly different extent (Figure 3b).^63,101,103^ Some *B. longum* strains degrade mono- and/or disubstituted AXOS and linear XOS, most likely making use of their extracellular ABFs and BXs (LMG 11047, CUETM 193, and CUETM 239), while others can only utilize arabinose released via extracellular ABFs, while BXs are absent (NCC2705, BB536, ATCC 15707 = LMG 13197, 46, CUETM 172, PRO 16–10, CUETM 290, and CUETM 171) (Table 2).^63,101–103^ Other *B. longum* strains only break down small XOS (DP < 4) likely with intracellular BXs (LMG 11588), and some cannot use AX hydrolysis products at all (LMG 13196, ATCC 51870, and LMG 11570).^63,101–103^ In contrast, Komeno et al.^102^ showed that the *B. longum* strain NCC2705, which could utilize arabinose from disubstituted AXOS,^63,103^ was not able to grow on purified disubstituted A^2+3^XX (Table 2). Supposedly, because of their varying AXOS utilization mechanisms and preference for specific AXOS structural elements, multiple *B. longum* strains may be involved in the cooperative utilization of AXOS in the gut. For example, *B. longum* strains that exclusively utilize arabinose from AXOS with extracellular ABFs produce linear XOS, which may be utilized by other *Bifidobacterium* strains that have the specific transporters to internalize these XOS.

The strain-dependent AXOS degradation in *Bifidobacterium* is likely exclusive to *B. longum* as it is the only species that shows an equally high variability of GH-encoding genes in their gene profile among strains (Figure S1).^26^ However, this gene profile only consists of strains reported in the CAZy database, in which not all published *Bifidobacterium* strains are available. For example, *B. adolescentis* LMG 10734 (not characterized in CAZy) was not able to utilize AXOS,^63^ though all other reported *B. adolescentis* strains in CAZy (10 total, including ATCC 15703) possess multiple different ABF (in GH43 and GH51), BX (in GH3, GH43, and GH120) and Rex (in GH8) encoding genes, including a GH43_10 that potentially hydrolyzes arabinosyl (Ara) from disubstituted xylosyl (Xyl) (Figure S1).^26^ Whether this discrepancy relates to the absence of AXOS-degrading genes in the genome of *B. adolescentis* LMG 10734, its inability to actually produce corresponding and active enzyme-proteins or the lack of suitable sugar transporters has to be further studied.

#### Complete AXOS utilization by B. adolescentis, B. catenulatum, and B. pseudocatenulatum

Strains that completely utilize AXOS include *B. adolescentis* ATCC 15703 (LMG 10502),^47,63,97,98^ *B. adolescentis* B72,^101^ *B. catenulatum* LMG 11043,^63^ and *B. pseudocatenulatum* YIT 4072^10^ (Table 2), which is in line with their genetic GH profiles indicating a wide variety and abundance of ABF (in GH43 and GH51), BX (in GH43, and GH120) and Rex (in GH8) encoding genes (Figure S1).^26^ In fact, how *B. pseudocatenulatum* YIT 4072 utilized various AXOS, was detailed in a comprehensive study by Saito et al. (2020), via transcriptome analysis and protein/enzyme characterization.^10^ They found that *B. pseudocatenulatum* YIT 4072 transported XOS and AXOS (mono- and disubstituted) into the cell via three ABC transporters with varying specificities (either narrow or broad), followed by the subsequent hydrolysis of these AXOS with intracellular BXs (GH43 and GH120), Rex (GH8) and ABFs (GH43).^10^ Interestingly, its GH51 genes (GH51_1 and GH52_2, Figure S1) were not upregulated upon AXOS supplementation, again indicating that genetic information is merely indicative and does not prove actual enzyme activity.^10,26^

In contrast to *B. pseudocatenulatum* YIT 4072, besides intracellular, also extracellular AXOS-degradation mechanisms have been suggested for *B. catenulatum* LMG 11043 and the *B. adolescentis* strains ATCC 15703 and B72.^8,63,101^ Based on their GH gene profiles and the product profiles from AXOS fermentation, *B. adolescentis* ATCC 15703 and B72 produce extracellular ABFs and BXs to degrade AXOS, and preferably grow on XOS (DP > 2) with limited growth on xylobiose, which, though not proven, could be related to the presence of XOS specific transporters with low binding affinity for xylobiose.^8,63,101^ The capability of *B. adolescentis* to fully degrade AXOS with extracellular enzymes while preferentially growing on specific substrates (i.e., XOS with DP > 2), suggests cooperation between various *Bifidobacterium* strains that utilize different parts of the AXOS, and may even stimulate strains that do not possess the enzymes to degrade AXOS yet grow on its degradation products, unlike the “selfish” AXOS utilization by *B. pseudocatenulatum* YIT 4072. Indeed, in complex microbiome communities, multiple *Bifidobacterium* species and possibly even strains are often stimulated together by AXOS, including *B. adolescentis*, *B. longum*, *B. catenulatum*, and *B. angulatum* (Table 1).^72,74–76^ Besides, *B. adolescentis* ATCC 15703 could not grow on substituted oligosaccharides from corn, indicating it does not have the machinery to degrade and utilize more complex substituted XOS compared to the relatively simple AXOS from wheat or rye.^94^ The upregulation of relevant GH encoding genes in these bacteria, and the location of the enzyme activity (i.e., intra- or extracellular), in combination with the presence of specific transporters, and thus the structure of the AXOS, can highly change the capability for these bacterial strains to grow on such AXOS.

#### Cross-feeding dependent AXOS utilization by Bifidobacterium

*B. angulatum*, *B. animalis*, *B. gallicum*, and *B. pseudolongum* can utilize the monosaccharides arabinose and xylose and potentially XOS, but did not grow on AXOS.^63,101^ This could be related to the relatively low amount of ABF encoding genes in these species, compared to *Bifidobacterium* species that degrade AXOS (e.g., *B. longum* and *B. adolescentis*) (Figure S1).^26^ These bacteria can potentially still be stimulated by AXOS,^72^ but require “help” from other “non-selfish” bacteria that produce extracellular ABFs and BXs, like for example *B. adolescentis*.

*B. bifidum*, *B. breve*, and *B. thermophilum* could not utilize any AX degradation products including arabinose and xylose.^63,101^ Indeed, they possess barely any AXOS-degrading enzymes (Figure S1),^26^ and possibly, but currently not known, lack corresponding transporters.

#### Metabolite profiles by Bifidobacterium

*Bifidobacterium* gains energy by degrading monosaccharides, via pyruvate, into acetate and lactate, through a metabolic pathway called the “bifid shunt”.^101,114,115^ The ratio of acetate and lactate produced depends on the substrate, species, and strain (Table 2).^101,114,115^ Nevertheless, some *B. longum* strains produce additional acetate, formate, and ethanol instead of lactate when metabolizing xylose and arabinose (Table 2).^101,103,115^ The formation of these other metabolic products instead of lactate could be caused by a difficulty to degrade and thus grow on the substrate for certain *Bifidobacterium* strains and is the result of a need for additional ATP production.^101,115^ Unexpectedly, Ríos-Ríos et al.^96^ and Sajib et al.,^100^ reported the formation of propionate and butyrate besides acetate and lactate by *B. adolescentis* ATCC 15703 from (A)XOS, and Bhattacharya et al.^95^ reported the formation of acetate, lactate, and propionate, but not butyrate by *B. adolescentis* ATCC 15703. The reported propionate and butyrate in these studies cannot be explained based solely on *Bifidobacterium* growth in particular, because *Bifidobacterium* does not possess the genes to produce propionate and butyrate.^107^ In complex microbiome studies, however, the stimulation of *Bifidobacterium* often coincides with the production of propionate and butyrate besides acetate and lactate, indicating a cross-feeding interaction and the stimulation of other gut bacteria by AXOS (Table 1).^7,70,76,84,103^ Nevertheless, lactate is often detected only during initial phases after AXOS supplementation, and decreases toward later stages (Table 1),^61,62,67–69^ likely because lactate is utilized more quickly by cross-feeders compared to acetate.^6,116^

### Lactobacillaceae

Recently, in 2020, the *Lactobacillus* genus was reorganized, and many species previously described as *Lactobacillus* were reclassified into many new genera in *Lactobacillaceae*.^93^ The stimulation of *Lactobacillus* by AXOS from previous studies (i.e., as in Table 1) will therefore be referred to as a stimulation of *Lactobacillaceae* in this review. Therefore, however, differences in stimulation of specific *Lactobacillaceae* genera and strains by AXOS are not reflected in this review, and should be addressed in future studies because of large genetic diversity in the *Lactobacillaceae* family.

*Lactobacillaceae* are often stimulated by higher DP AXOS (DP > ~10) in complex microbiome communities (Table 1).^65,69,80,84,92^ Though often stimulated, *Lactobacillaceae* are not consistently stimulated by higher DP AXOS in all studies (Table 1),^67,68,77^ and in one case also lower DP AXOS (DP = 3 to 8) caused a significant increase in *Lactobacillaceae*.^73^ Variation among studies is likely because *Lactobacillaceae* is only present in low abundance in the human colon,^6^ *Lactobacillaceae* is a heterogenous family with large genetic diversity,^117^ and gut microbiomes are complex communities where differences in bacterial composition may either facilitate or hinder the growth of *Lactobacillaceae* (through cross-feeding or competition). To further confirm the preferential growth of *Lactobacillaceae* on higher DP AXOS, Geraylou et al.^65,92^ found that AXOS with an average DP of 32 significantly increased *Lactobacillaceae* in sturgeons, whereas AXOS with an average DP of 3 did not. When lower DP AXOS are directly supplemented to complex microbiome communities, *Lactobacillaceae* are often outcompeted by *Bifidobacterium* (Table 1).

#### Cross-feeding dependent AXOS utilization by Lactobacillaceae

Although stimulated by (high DP) AXOS in complex microbiome studies as mentioned above, most *Lactobacillaceae* strains barely possess any genes encoding AXOS-degrading enzymes (Figure S2),^26^ and currently no studies report the successful growth of any *Lactobacillaceae* species from AXOS in single culture studies.^96,97,99,100,118,119^ For instance, Ríos-Ríos et al.^96^ reported 10-fold less growth by *Lacticaseibacillus rhamnosus* BIO5326 on low DP AXOS (DP < 6) compared to *B. adolescentis* ATCC 15703, and indeed, BIO5326 and other *Lacticaseibacillus* species possess no genes encoding AXOS-degrading enzymes (Figure S2).^26^

*Levilactobacillus brevis* is one of the few species in *Lactobacillaceae* (besides *Levilactobacillus koreensis*, *Schleiferilactobacillus harbinensis*, *Lentilactobacillus buchneri*, and *Paucilactobacillus oligofermentans*) that does contain a substantial number of genes encoding AXOS-hydrolyzing enzymes (BXs in GH3 and GH43, Rex in GH8 and ABFs in GH51 and GH43) (Figure S2).^26,99^ However, despite of their ABF genes, *L. brevis* DSM 20,054 and DSM 1269 could not grow on AXOS, yet did grow well on the monosaccharides arabinose and xylose and linear XOS,^97,99,100,118^ indicating that, as also found for certain *Bifidobacterium* strains, the presence of certain genes encoding fiber-degrading enzymes does not guarantee their upregulation and activity. Instead, in complex microbiome communities, these *Lactobacillaceae* likely cross-feed off XOS, arabinose, and xylose, which are released by other gut bacteria that produce extracellular ABFs, BXs, and xylanases. Indeed, *Lactobacillaceae* have been found to be co-stimulated by AXOS together with either *Bifidobacterium*, Bacteroidales, and/or Clostridia that potentially produce these enzymes in multiple studies (Table 1).^65,69,73,80,84,92^ The dependency of *Lactobacillaceae* on other gut bacteria for their growth on AXOS may reflect their inconsistent growth among individuals (i.e., as seen in Table 1), and should be kept in mind when supplementing AXOS as prebiotic to target specific *Lactobacillaceae*.

#### Metabolite profiles by Lactobacillaceae

*Lactobacillaceae* that can utilize arabinose and xylose, metabolize these pentoses, via intermediates, to predominantly lactate and acetate in equal molar amounts through the phosphoketolase pathway or to only lactate via the pentose phosphate pathway.^69,97,100,120–122^ The production of similar metabolic products compared to *Bifidobacterium* (i.e., acetate and lactate) is in line with a similar stimulation of cross-feeders and a similar production of acetate, propionate, and butyrate in complex microbiome studies (Table 1).^65,69,73,80,84,92^

### Bacteroidales

The Bacteroidales genera *Bacteroides* and *Prevotella* grow on AXOS, seemingly irrespective of their structure and thus are able to grow on low and high DP AXOS from various sources (Tables 1 and 2).^68–70,73,74,80,84,85,96,98,104,106^ Unfortunately, multiple studies could not pin-point an exact increase of either *Bacteroides* or *Prevotella* due to limitations in the analysis method used, and could only quantify their combined increase (Table 1).^68,69,80,84^ Furthermore, multiple *Bacteroides* species were recently reclassified as *Phocaeicola*, including the abundant plant-saccharide degrading gut bacteria *P. vulgatus* and *P. dorei*.^98,123–125^ Because of a lack of accurate identification of which Bacteroidales species are stimulated by AXOS in these complex microbiome studies (Table 1), it is still unknown which exact species are the more relevant AXOS degraders and future growth studies should therefore be analyzed with today’s more sophisticated techniques of microbiome analysis.

#### GH gene profiles within Bacteroidales

Bacteroidales, especially *Bacteroides*, are known to be active on a large variety of carbohydrate substrates, not limited to arabinoxylan and AXOS.^94,126^ As expected from their capability to grow on various (low and high DP) AXOS (Table 1), *Bacteroides* and *Prevotella* possess a large variety and abundance of genes encoding AXOS-degrading enzymes with ABFs in GH43 and GH51, BXs in GH3, GH39, and GH43 (and GH120 only for *Bacteroides*), and xylanases in GH10, GH5_21, and GH8 (which could also be a Rex). The recently reclassified *Phocaeicola* possess relatively few AXOS-degrading enzymes compared to *Bacteroides* and *Prevotella*, though they still have ABFs in GH43 and GH51, BXs in GH3, GH39, and GH43 and a potential xylanase in GH10 (Figure S3, S4 and S5).^26^ Though the presence of GH genes does not guarantee their upregulation and activity, still, the variety and abundance of such genes may be indicative for their growth on a certain substrate. The genes for AXOS utilization in these bacteria, i.e., the intra- and extracellular fiber-degrading enzymes and specific sugar transporters for the internalization of specific AX hydrolysis products, are clustered together in so-called polysaccharide utilization loci (PULs).^94,104,126^

#### Competition dictates the proliferation of Bacteroidales in the gut

Due to the large variety of AXOS-degrading enzymes in their genomes, *Bacteroides* and *Prevotella* are expected to grow on many types of AXOS structures, and because of their nonselective growth, *Bacteroides* and *Prevotella* likely occupy a similar metabolic niche in the gut. A comprehensive study by Chung et al.^74^ reported a negative correlation between the stimulation of *Bacteroides* and *Prevotella* across individuals consuming AXOS, implying that the proliferation of specific Bacteroidales genera is dictated by competition. Individuals harboring *Prevotella* in their initial microbiome composition before treatment showed an increase of *Prevotella* accompanied with a reduction of *Bacteroides*, whereas *Prevotella*-negative individuals showed an increase of *Bacteroides*.^74^ Hence, *Prevotella* may outcompete *Bacteroides* in the consumption of AXOS, but more studies will need to confirm this with above said techniques to distinct both *Bacteroides* and *Prevotella*.^74^ Another study, with only three fecal donors, showed that *Prevotella* and *Bacteroides* were also selectively stimulated by AXOS in two donors (i.e., either only *Bacteroides* or only *Prevotella*), though, in contrast, in one donor *Bacteroides* and *Prevotella* were stimulated simultaneously (Table 1).^70^ Further corroborating the expected competition among Bacteroidales in the gut is the proposed division of the human gut microbiota into three enterotypes, which are either dominated by *Bacteroides*, *Prevotella*, or *Ruminococcus*.^127,128^ The competition among species within these genera is dependent on the specific strains that are present in the gut and their specific AXOS utilization mechanisms,^126^ and thus is expected to vary among individuals.

#### AXOS utilization by Bacteroides

Though GH genes are not the only relevant indicators for a specific species’ competitive growth on a certain substrate, the variety and abundance of GH genes are still indicative, and knowledge on transporters and intra- or extracellular AXOS degradation mechanisms within bacteria is still limited. Therefore, based on variety and abundance of potential AXOS-hydrolyzing GH genes (see text above, Figure S3), the most relevant AXOS-degrading *Bacteroides* species likely include *B. intestinalis*, *B. cellulosilyticus*, *B. oleiciplenus*, *B. ovatus*, *B. xylanisolvens*, and *B. stercorirosoris*. Indeed, *B. intestinalis*, *B. cellulosilyticus*, *B. ovatus*, and *B. xylanisolvens* grew on many types of AXOS, including mono- and disubstituted AXOS (Table 2).^98,106^ Furthermore, the utilization of arabinoxylan by *B. ovatus* ATCC 8483 has been investigated in detail through protein/enzyme characterization by Rogowski et al.^94^ They found that *B. ovatus* ATCC 8483 degrades simple AX from wheat with surface-located extracellular GH10 endoxylanases, GH3 BXs, and GH43 ABFs that cleave Ara from monosubstituted Xyl.^94^ (A)XOS are then transported into the periplasm where they are further degraded with GH10 endoxylanases and GH43 ABFs active on both mono- and disubstituted Xyls, and the resulting low DP linear XOS are then transported into the cytoplasm and degraded with a GH43 BX.^94^ Other enzymes and transporters were involved in the degradation of more complex substrates like corn arabinoxylan.^94^ Although the AX degradation machinery of *B. ovatus* ATCC 8483 is optimized for “selfish” intracellular degradation of oligosaccharides, it was able to support the growth of *B. adolescentis* ATCC 15703 through the production of (A)XOS via the degradation of AX with its extracellular GH10 endoxylanase.^94^ Therefore, *B. ovatus*, and potentially other *Bacteroides* species, are expected to also facilitate cross-feeding of *Bifidobacterium* and *Lactobacillaceae* from (high DP) AXOS with extracellular enzymes. Still, the surface located extracellular GH10 and sugar transporters are not present in all *B. ovatus* strains,^94^ and beyond a species dependency in *Bacteroides*, the AXOS-degradation may also be strain dependent, as found for *Bifidobacterium*. *B. eggerthii* grew on the same AXOS as the other above mentioned *Bacteroides* species, yet to lesser extent (Table 2),^106^ which could be related to a lesser abundance and variety of GH genes relevant for AXOS degradation in *B. eggerthii* strains (Figure S3).^26^ *B. thetaiotaomicron* ATCC 29148 showed no activity on most AXOS, it did, however, grow on free arabinose, xylose, xylobiose, and A^3^X (Table 2),^106^ yet *B. thetaiotaomicron* strains possess a large amount of ABF encoding genes found in GH43 and GH51 (Figure S3).^26^ *B. thetaiotaomicron* ATCC 29148 could thus potentially, though not proven, take up small (A)XOS with a backbone of one or two Xyls and degrade them with intracellular ABFs and BXs. Though these *Bacteroides* have such a high abundance and variety of GH encoding genes (Figure S3),^26^ genetic differences among species and strains resulting in lack or presence of specific AXOS utilization machinery could dictate the competitiveness among the *Bacteroides* genus.

#### AXOS utilization by Prevotella

In *Prevotella*, again based on variety and abundance of potential AXOS-hydrolyzing GH genes (see text above, Figure S4), the most relevant AXOS-degrading species likely include *P. copri*, *P. bryantii*, *P. ruminicola*, *P. herbatica*, *P. paludivivens*, *P. oryzae*, *and P. albensis*. Indeed, *P. copri* DSM 18205 grew well on AX from barley and on AXOS from barley and wheat,^96,104^ and *P. copri* was stimulated by AXOS in complex microbiome communities.^72,74^ Hence, the *P. copri* species is likely a relevant competitor in AXOS utilization among the Bacteroidales order. Though no complete biochemical characterization of the AXOS utilization machinery has been performed in *P. copri* so far, one extracellular xylanase in GH10 and an extracellular ABF in GH43 have been characterized in *P. copri* DSM 18205.^104^ Therefore, similar to *Bacteroides*, these *Prevotella* may also facilitate cross-feeding of *Bifidobacterium* and *Lactobacillaceae* from (high DP) AXOS via their extracellular fiber-degrading enzymes. Another *Prevotella* strain, *P. ruminicola* 23, grew on purified acetylated/feruloylated AXOS from corn via upregulation of carbohydrate esterases (Table 2),^105^ indicating that not only *Bacteroides* but also *Prevotella* may degrade many different AXOS structures using their large variety of fiber-degrading enzymes.

#### AXOS utilization by Phocaeicola

*Phocaeicola* have less variety and abundance of GHs in their genome compared to *Bacteroides* and *Prevotella* (see text above, Figure S5); still, the most relevant AXOS-degrading species likely include *P. dorei* and *P. vulgatus* (Figure S5).^26^ Indeed, *P. vulgatus* ATCC 8482 completely degraded AXOS from wheat flour likely based on its ABFs and BXs, but did not degrade AX,^98^ although it has a GH10 encoding gene (Figure S5).^26^ *P. dorei* DSM 17855 also possesses a GH10 encoding gene (Figure S5),^26^ and still expressed no xylanase activity on birchwood and oat spelt xylans.^129^ As shown previously, presence of certain GH genes does not guarantee their upregulation and activity. *B. ovatus* ATCC 8483 also possesses an inactive GH10 encoding gene, which was shown to be a xylan binding protein,^94^ hence, this GH10 gene in *Phocaeicola* may also be responsible for a specific transporter binding affinity. Still, the utilization of AXOS by *Phocaeicola* has not yet been properly investigated, and, unlike for *Bacteroides* and *Prevotella*, it is currently unknown if *Phocaeicola* are relevant competitors in the utilization of AXOS in complex gut microbiome communities.

#### Metabolite profile by Bacteroidales

Bacteroidales mainly generate propionate, acetate, and succinate (besides trace amounts of lactate and formate) from carbohydrates through the “succinate pathway”.^130–132^ In the succinate pathway, acetate and succinate are produced from monosaccharides, and subsequently, the produced succinate may be further metabolized to propionate.^130–132^ Indeed, a stimulation of Bacteroidales by AXOS in complex microbiome studies coincided with an increase in acetate and propionate in multiple studies (Table 1).^68–70,72,80,84,85^ Nevertheless, not all species in Bacteroidales possess the enzyme to generate propionate from succinate, and thus mainly produce acetate and succinate (for example, *P. brevis* GA33 and *P. vulgatus* ATCC 8482),^131,133,134^ which could be utilized by cross-feeding bacteria. Surprisingly, besides acetate and propionate, butyrate production was reported for *P. copri* DSM 18205 from AXOS (Table 2),^96^ which was unlikely to be true because the *P. copri* genome does not possess any known butyrate production pathways.^107^

### Clostridia

Clostridia (predominantly from the *Lachnospiraceae* and *Oscillospiraceae* families) are butyrate-producing cross-feeders that utilize the metabolites acetate and lactate produced by other gut bacteria.^7,135^ In complex gut microbiome communities, AXOS stimulated specifically *Agathobacter*, *Roseburia*, *Faecalibacterium*, *Anaerostipes*, *Eubacterium*, *Lachnoclostridium*, *Oscillospira*, *Blautia*, *Ruminococcus*, *Dorea*, and *Fusicantenibacter* (Table 1).^65,70–72,76,85,91,92^ Though in some cases a reduction of *Faecalibacterium*, *Eubacterium*, *Oscillospira*, *Blautia*, *Ruminococcus*, or *Dorea* has been reported as well (Table 1).^70,71,73^ Overall, which specific Clostridia are increased or decreased highly varies among studies and individuals (Table 1).

#### Metabolite utilization by Clostridia

The major butyrate producing Clostridia in the human gut include *Faecalibacterium prausnitzii*, *Agathobacter rectalis*, *Anaerobutyricum hallii*, *Roseburia*, *Anaerostipes*, and *Butyricicoccus*.^7^ These Clostridia produce butyrate from acetate, by converting two acetyl-coenzyme A (CoA) molecules into butyryl-CoA followed by the transfer of CoA to an exogenous acetate through the butyryl-CoA:acetate CoA-transferase route.^7,116,136^ *Anaerobutyricum hallii* and *Anaerostipes* spp. can also produce butyrate from lactate, by conversion of lactate to acetyl-CoA, which binds an exogenous acetate to form butyrate.^7,137^ Therefore, these butyrate-producers flourish in co-occurrence with acetate and lactate producing *Bifidobacterium* or *Lactobacillaceae* (Table 1). Because these Clostridia cross-feed off the metabolites produced by other gut bacteria that degrade AXOS, their proliferation in the gut is likely more dependent on the specific species or strains that are present in the gut, more so than on the specific AXOS structures supplemented. Interestingly, *Roseburia* and *Agathobacter rectalis*, and possibly other Clostridia species, are also active as degraders of AX and AXOS and can ferment these substrates to predominantly butyrate.^95,103,113^

#### AXOS utilization by Roseburia

Though only few *Roseburia* strains are characterized in CAZy, based on the available genome data, the most relevant AXOS utilizing strains are found in *R. intestinalis* and *R. hominis* (Figure S6).^26^ Some strains in *R. intestinalis* possess a potential GH10 xylanase encoding gene, which is expected to cause a major difference in growth on AX and AXOS compared to other *Roseburia* strains that lack this gene (Figure S6).^26^ One of such strains is *R. intestinalis* L1–82, which, through transcriptome analysis and protein/enzyme characterization, was proven to degrade AX with an extracellular endoxylanase (GH10) followed by the intracellular degradation of AXOS with ABFs (GH43 and GH51), BXs (GH3, GH39, and GH120) and Rex (GH8) after uptake of AXOS (DP > 4) via ABC transporters.^113^ The currently only characterized *R. hominis* strain in CAZy (DSM 16839) does not possess endoxylanases in its genome (Figure S6),^26^ yet did grow on AXOS from wheat (Table 2),^95^ likely based on its’ ABFs (GH43 and GH51) and BXs (GH3 and GH43) (Figure S6).^26^ Nevertheless, though these *Roseburia* possess the machinery to degrade and utilize AXOS, only one complex microbiome study has reported a significant increase of *Roseburia* by AXOS (Table 1),^70^ which suggests that *Roseburia* are often outcompeted by other gut bacteria from AXOS, like, for example, *Bifidobacterium*.

#### Acetate-dependent AXOS utilization by Agathobacter rectalis

*A. rectalis* ATCC 33656 was able to grow on AXOS, but only in the presence of acetate (Table 2).^103^ Acetate is required as co-substrate for *A. rectalis* to grow on AXOS,^103^ and this acetate dependence has been demonstrated for strains in *Roseburia*, *Anaerobutyricum hallii*, *Faecalibacterium prausnitzii* as well when growing on inulin-type fructans.^7^ However, acetate as co-substrate is not required for the *Roseburia* strains described above when growing on AX and AXOS,^95,113^ hence, this acetate-dependence may be genus and/or substrate specific among fiber-degrading Clostridia. *A. rectalis* ATCC 33656 consumed acetate and simultaneously released the monosaccharides xylose and arabinose from AXOS, likely based on its extracellular ABFs (in GH43 and GH51) and BXs (in GH3 and GH43), accompanied with the formation of butyrate (Table 2, Figure S6).^26,103^ *A. rectalis* could also utilize the released monosaccharides, but only after all oligosaccharides were degraded.^103^ *A. rectalis* are thus predominantly cross-feeders that utilize acetate (and AXOS) to produce butyrate, and simultaneously facilitate cross-feeding of bacteria that do not possess the enzymes to fully degrade AXOS, which was confirmed by the successful co-culture growth of *A. rectalis* ATCC 33656 and *B. longum* NCC2705 on AXOS (Table 2).^103^

#### Other potential AXOS-degrading Clostridia

*Ruminococcus*, *Blautia*, and *Eubacterium* were also significantly increased after supplementation of AXOS in complex microbiome communities (Table 1),^65,70,72,76,92^ and, depending on the species, may potentially be active as degraders of AXOS based on the presence of ABF, BX, and xylanase encoding genes in these genera (Figure S6).^26^ Nevertheless, the presence of these genes does not guarantee their corresponding enzyme activity and disregards other important AXOS utilization mechanisms like sugar transporters and even the competitiveness compared to other gut bacteria. Though the presence of these GH genes may still be indicative, and based on their available GH gene data, the most relevant AXOS-degrading species could potentially be found in *R. albus* and *R. flavefaciens*, *E. xylanophilum*, *E. uniforme*, *E. ruminantium*, and *E. cellulosolvens*, *B. producta*, *B. coccoides*, and *B. pseudococcoides* (Figure S6).^26^ Whether these bacteria are actually active as AXOS degraders or cross-feeders, or both, has not yet been studied and needs further research. Though *Blautia* have been shown to be active as cross-feeders that produce acetate from H_2_, CO, and CO_2_.^138,139^

*Faecalibacterium*, *Dorea*, and *Anaerostipes* hardly possess any AXOS-degrading enzymes and proliferate predominantly through cross-feeding off secondary metabolites (Figure S6).^7,26^ Unexpectedly, *F. duncaniae* DSM 17677 reportedly grew well on AXOS and produced predominantly propionate (Table 2),^96^ though this is unlikely to be true because *F. duncaniae* DSM 17677 does not possess any known genes for AXOS degradation or propionate production (Figure S6).^26,107–109^

### Negativicutes

Negativicutes lack AXOS-degrading enzymes (CAZy.org),^26^ and are cross-feeders that utilize the metabolic products from other AXOS degraders in the gut. The genera *Megamonas*, *Veillonella*, *Selenomonas*, *Dialister*, *Megasphaera, Acidaminococcus*, and unclassified *Erysipelotrichaceae* were significantly increased by AXOS (Table 1).^70,71,84,85^ Similar to Clostridia, the stimulation of specific Negativicutes genera via AXOS highly varies among studies and individuals (Table 1). The metabolites they can utilize and produce also vary among genera, for example, *Veillonella* produce acetate and propionate from lactate,^140,141^ *Selenomonas* produce succinate and propionate from lactate,^84,142,143^ *Dialister* produce propionate from succinate,^144,145^ and *Megasphaera* produce butyrate from acetate or lactate.^142,143,146,147^ Still, the utilization of metabolic products from AXOS fermentation and thereby the impact of these Negativicutes on the gut has not yet been properly investigated, and requires more research.

## Conclusion

Arabinoxylan hydrolysis products, also known as arabinoxylo-oligosaccharides (AXOS), impact the gut microbiome in a structure-dependent matter, by stimulating specific AXOS-degrading and cross-feeding bacterial species. The AXOS are degraded by primary and secondary degraders comprising *Bifidobacterium*, *Lactobacillaceae*, Bacteroidales, and Clostridia, of which the specific species and strains that are stimulated are dependent on the cooperation and competition between these bacteria. Their specific AXOS fermentation mechanisms (i.e., presence of intra- or extracellular AXOS-degrading enzymes and sugar transporters) vary vastly among different genera, species, and even among strains, resulting in either a “selfish” or a “synergistic” stimulation of certain gut bacteria, depending not only on the AXOS structure but also the individual’s gut microbiome composition.

Based on our literature review and novel *in silico* analysis, we propose that a promising future perspective is to aim for a defined and selective prebiotics package, of which the exact nature will have to highly correlate with the individual’s gut microbiome profile. To make such an aim a reality, metagenomic and transcriptomic data of the gut microbiome will be leading to define prebiotics packages, to support specific beneficial bacterial strains with targeted prebiotics. In addition, combinations of carefully selected pre- and probiotics (e.g., synbiotics) can fill gaps in individual’s gut microbiomes. Overall, our review already provides specific information on promising combinations of AXOS structures and strains as leads for precision prebiotics. Further research subjecting specific prebiotic structures to single- and multiculture fermentations, fecal fermentation studies, and *in vivo* trials analyzed with today’s sophisticated techniques for accurate microbiome profiling will need to confirm the effectiveness of these leads to modulate the individual gut microbiome.

## Supplementary Material

Supplemental Material

## Data Availability

There is no new research data generated in this paper.
