# Supplementary figures and images for: Structure-dependent stimulation of gut bacteria by arabinoxylo-oligosaccharides (AXOS): a review
Source: Gut Microbes. 2024 Nov 29;16(1):2430419. doi: 10.1080/19490976.2024.2430419 (PMC11610566; doi:10.1080/19490976.2024.2430419)

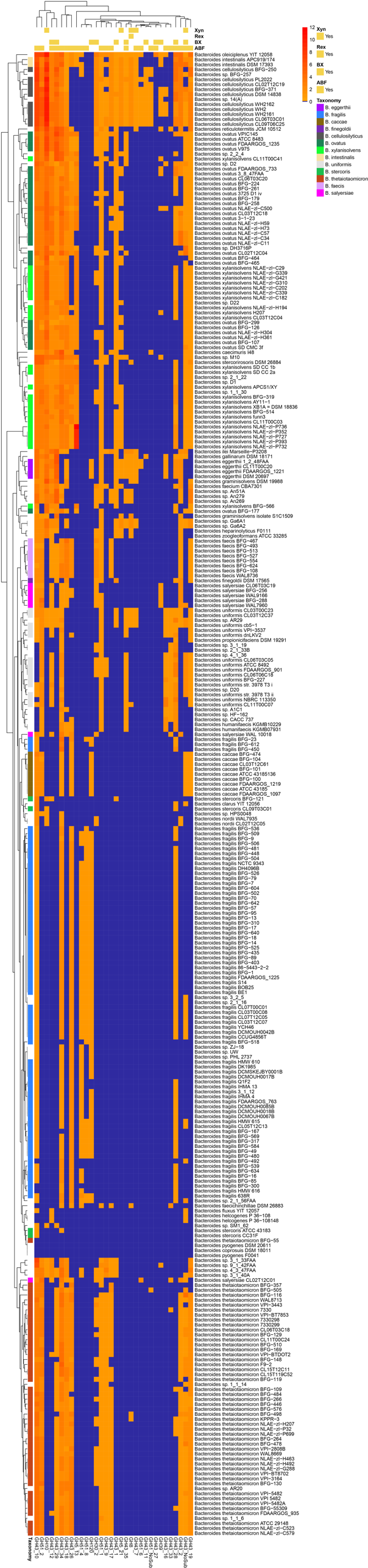

Supplement: Supplemental Material [file KGMI_A_2430419_SM7662.zip › Figure S3 Bacteroides.png]

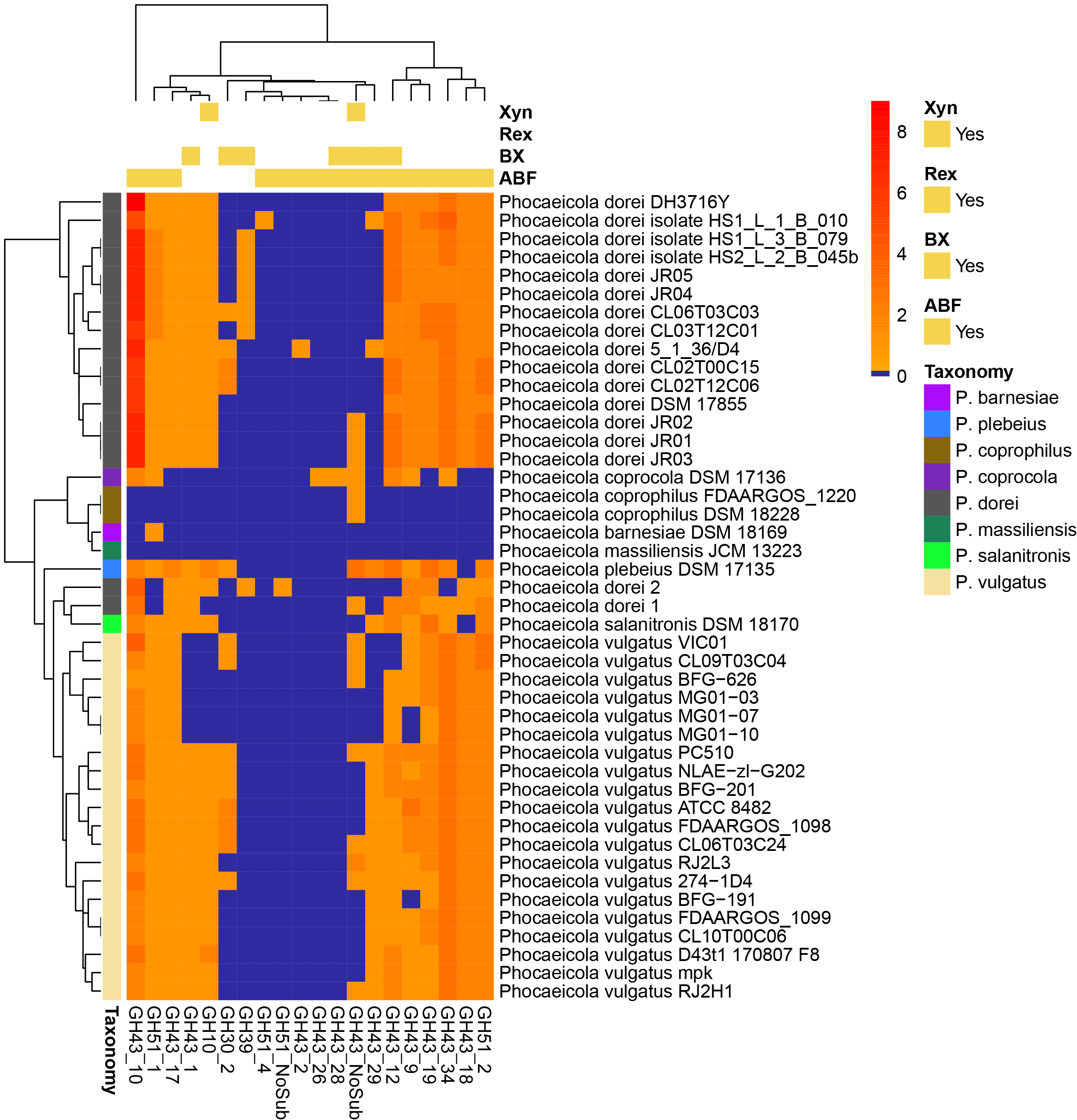

Supplement: Supplemental Material [file KGMI_A_2430419_SM7662.zip › Figure S5 Phocaeicola.png]

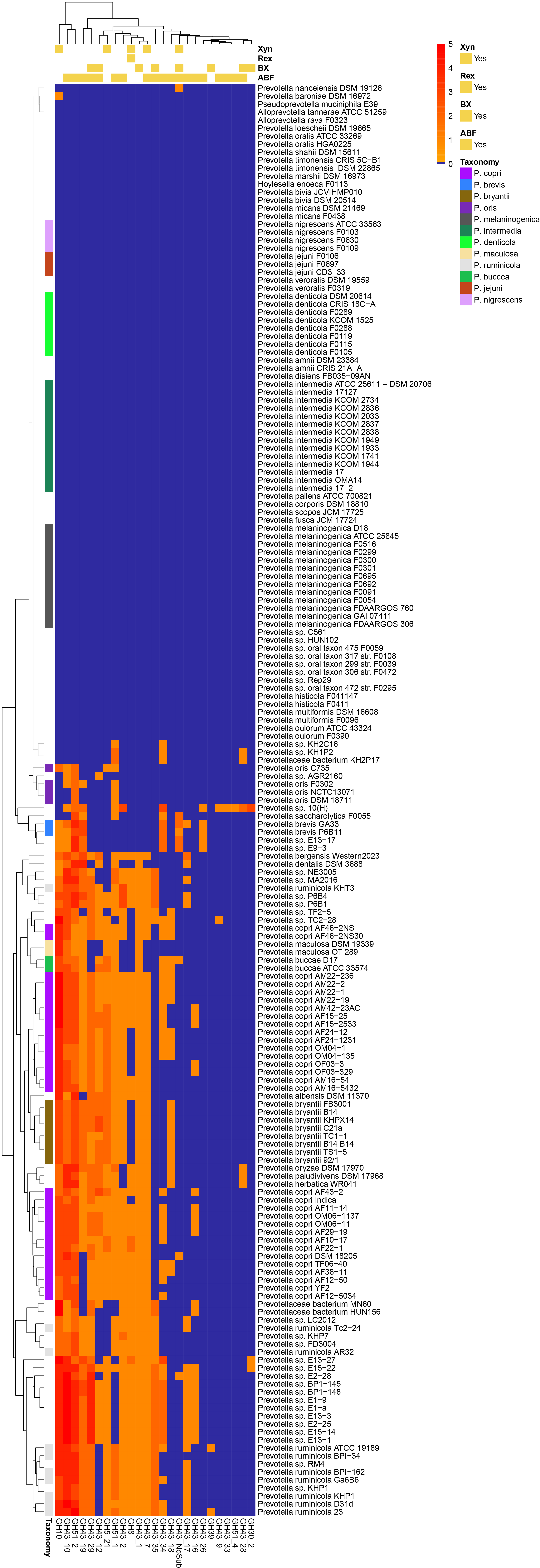

Supplement: Supplemental Material [file KGMI_A_2430419_SM7662.zip › Figure S4 Prevotella.png]

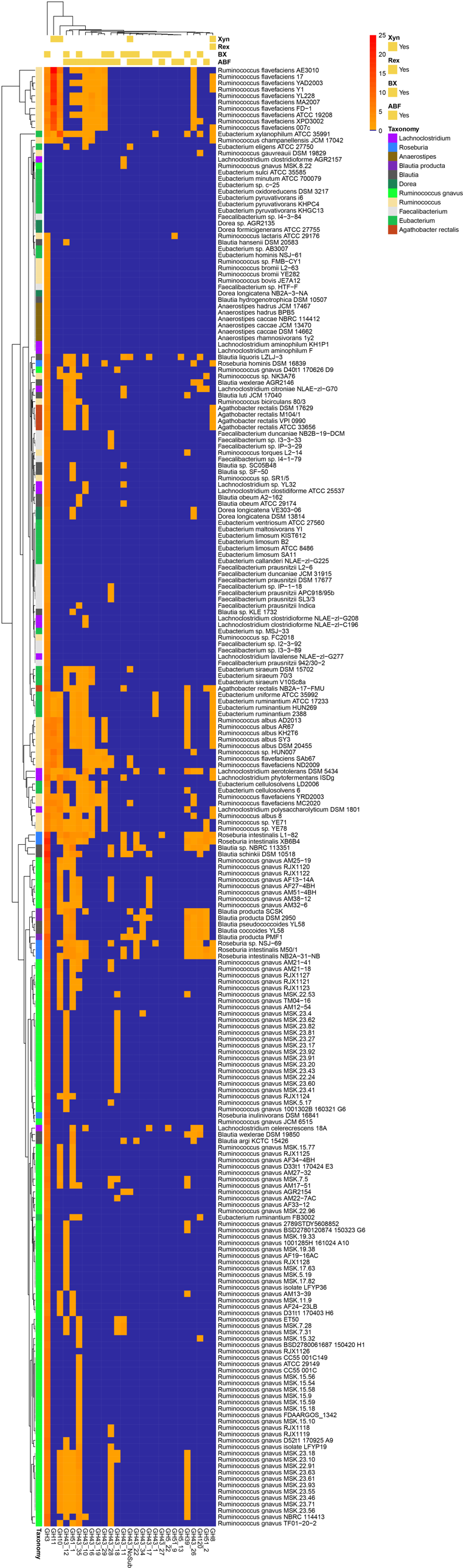

Supplement: Supplemental Material [file KGMI_A_2430419_SM7662.zip › Figure S6 Clostridia.png]

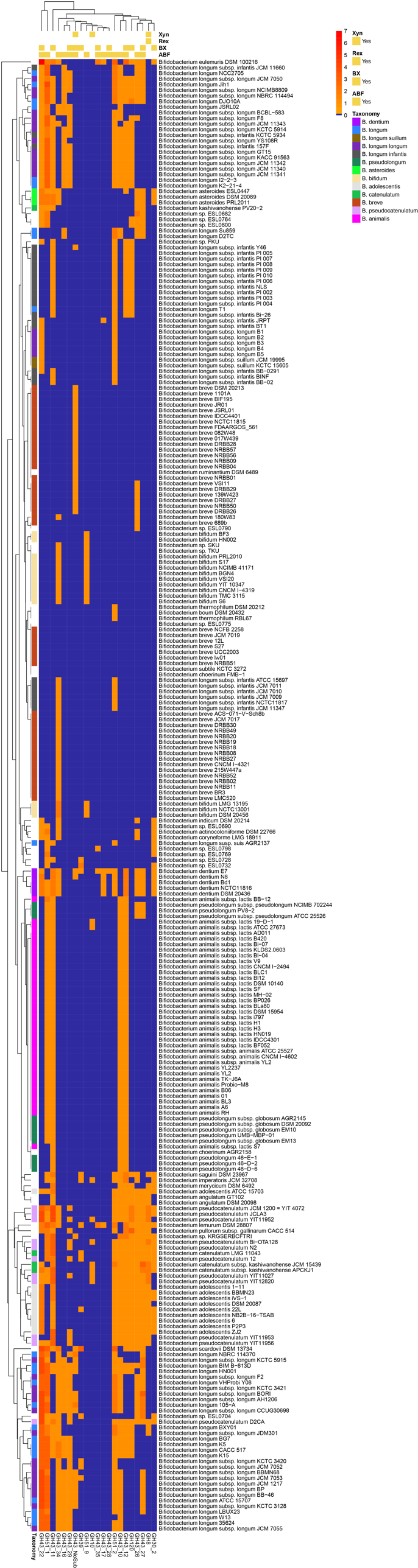

Supplement: Supplemental Material [file KGMI_A_2430419_SM7662.zip › Figure S1 Bifidobacterium.png]
